# Audit of CTO Reports in a Community Mental Health Team

**DOI:** 10.1192/bjo.2026.11747

**Published:** 2026-06-30

**Authors:** Segun Ayanda, Manhar Randhawa, Saira Bano, Emmanuel Oranusi, Roger Singh

**Affiliations:** KMMH, Medway, United Kingdom

## Abstract

**Aims:**

Community Treatment Orders are legal powers within community mental health services designed to support patient compliance with medication and overall continuity of care while maintaining the least restrictive principles of care. Their effective use, however, is dependent not only on effective clinical decision-making but also on accurate and timely completion of Community Treatment Order reports, which are essential for tribunal reviews and renewal decisions. Capacity assessments regarding whether the patient can consent to such a legal process are another important step in safeguarding the patient’s human rights, and their completion is fundamentally essential to the whole process.

In practical reality, however, the Community Treatment Order process is often experienced by doctors and nurses as administratively complex, with unclear processes and procedures, and reports frequently required in a time-pressured fashion. This can lead to breaches of legal requirements for reviews. MHT Medway is an example of this, where concerns were raised about delays in report completion rates by locality, leading to tribunal delays, increased clinician workload, and governance concerns. This prompted a retrospective audit to understand the status quo and initiate a system change.

**Methods:**

A twelve-month retrospective audit covering January 2025 to January 2026 was conducted across the three localities in Medway Community Mental Health Team (Gillingham, Chatham, and Rochester). Anonymised data collected included the Community Treatment Order start date, report completion status, completion date, and type of report submitted (full medical report or addendum), capacity forms to consent, the clinician who completed the report, and the responsible clinician for each patient. Data were collected manually by reviewing physical and digital records and entered into an Excel spreadsheet for analysis.

**Results:**

There appeared to be variation in report completion rates across localities, withChatham at 66.7%, Gillingham at 73.3%, and Rochester at 33%. Report completion was unevenly distributed across the months, with clustering around periods when reviews were due. Full medical reports accounted for 74% of completed reports, addendum reports accounted for 16%, and the remainder were combined reports.

In more than two-thirds of cases, capacity forms appeared to have been completed, with rates ranging from 100% in Rochester to 66% in Chatham. Of the six clinicians in the team, only three were completing reports, accounting for approximately 100% of completions. Completion rates were 73% and 50% across the two responsible clinicians. Access to previous reports was inconsistent. Physical and digital copies of current documentation were not available for some patients, and communication between localities regarding current caseload lists was limited.

**Conclusion:**

This audit highlighted system-level variation in medical report completion, clinician and locality differences in completion rates, and timing of reports being closely linked to review dates. These findings informed the implementation of a centralised communication system, improved documentation access, and enhanced administrative processes. A further audit is planned to evaluate the impact of the intervention.

